# Understanding the Barriers and Enablers for Seeking Psychological Support following a Burn Injury

**DOI:** 10.3390/ebj4030028

**Published:** 2023-07-24

**Authors:** Lianne McDermott, Matthew Hotton, Anna V. Cartwright

**Affiliations:** 1 Oxford Institute of Clinical Psychology Training and Research, Isis Education Centre, Warneford Hospital, Headington, Oxford OX3 7JX, UK; 2 Burns Unit, Stoke Mandeville Hospital, Buckinghamshire Healthcare NHS Trust, Aylesbury HP21 8AL, UK

**Keywords:** burns, psychological, burns unit, barriers, enablers, COM-B

## Abstract

Burn injuries can be traumatic and distressing for patients, with a prolonged period of recovery. This qualitative study aimed to explore adult burn patients’ perceptions of the barriers and facilitators to accessing psychological support in a Regional Burns Service in Southeast England. Participants (five females and six males) were under the care of the burns unit and were not currently accessing psychological support. Eleven semi-structured interviews were conducted. Responses were analysed using thematic analysis. Four main themes highlighted how access to psychology was influenced by communication between the patient and service, beliefs about mental health, environmental challenges, and patient hope. Recommendations for improving access to burn psychological care included (1) the provision of patient resources to increase awareness and reduce stigma; (2) psychological skills training to encourage staff to recognise distress and respond appropriately; (3) staff training in the practice of cultural humility; (4) increasing psychological presence in outpatient appointments and via routine follow-ups.

## 1. Introduction

The impact that sustaining a burn injury has on the physical and psychological domains of an individual’s life has been well documented [1,2]. Psychological concerns include adjusting to physical changes, post-traumatic stress disorder [3], anxiety and depression [4], pain [5], sleep disturbance [6], body image concerns [7], difficulties associated with intimate relationships, and/or an exacerbation of pre-existing psychological difficulties [8]. Untreated psychological distress post-burn injury has been associated with prolonged physical recovery [2]. A meta-analysis found rates of PTSD ranged from 3 to 35% at 1 month, 2 to 40% at 3–6 months, 9 to 45% at 1 year, and 7 to 25% at 2+ years post-injury [3]. The psychological effects of a burn injury are described to persist for years beyond the injury [9,10]. Although psychological concerns are prevalent across burn patients, there are several risk factors that may contribute to the development of psychopathology, including premorbid mental health disorders, alcohol misuse, and suicidality [11]. Identifying the premorbid psychological symptoms before and after a burn injury is therefore important for optimizing recovery. There is a need to better understand patients’ experiences during burns rehabilitation to improve access to psychological support and facilitate psychological recovery.

Michie et al.’s model suggests behaviour is influenced by three factors: capability, opportunity, and motivation [12]. In applying this to burn psychological help-seeking, an individual’s ‘capability’ to engage in support could be influenced by psychological factors. For example, research has shown that PTSD following a burn injury can be triggered beyond the scope of the incident itself [13], and invasive medical procedures can increase trauma symptoms [14,15]. The Cognitive Model of PTSD [16] posits that individuals who are ‘reminded’ of a traumatic events experience negative somatic, cognitive, affective symptoms, leading to avoidance. The psychology team is part of the Burns MDT, and therefore may serve to ‘remind’ patients of their traumatic experience, prompting avoidance. Second, the ‘opportunity’ to access psychology may be influenced by environmental factors. This has been evidenced by parents of burn-injured children, who report financial, time, and geographical barriers to seeking help [17]. Such barriers may be present for patients who have experienced a loss of physical/financial independence post-burn injury. Third, the ‘motivation’ to engage in psychological support may be influenced by beliefs about mental health [18]. Research has demonstrated that complex cultural frameworks inform individual health beliefs and symptom expression [19,20]. Taken together, it is important to understand how patients conceptualise psychological symptoms and their beliefs about the usefulness, and availability, of psychological treatment in this context.

### 1.1. Service Context

This project took place within a specialist NHS burns unit in South-East England. The unit treats adult patients who have experienced a burn injury below the level of 40% total body surface area. The multi-disciplinary team (MDT) consists of surgeons, nurses, occupational therapists, physiotherapists, psychologists, and dieticians. Within the psychology team, there are two part-time Clinical Psychologists and one full-time Assistant Psychologist. In line with the National Burns Care Standards (NBCS), the psychology team aim to screen all inpatients who are admitted for more than 24 h as soon as clinically appropriate and prior to discharge [21]. Across the UK, it is predominantly accepted that screening during a patient’s admission should consider the following domains: depression, anxiety, acute stress symptoms, PTSD, distress related to pain or appearance, and, risk of self-harm and suicidality [22]. Patients who are identified as experiencing psychological difficulties during screening are then offered further psychological assessment with a Clinical Psychologist, and psychological interventions are provided if appropriate. Should screenings highlight manageable levels of distress, patients and their families are provided with the burns unit’s contact details and informed they can self-refer for psychological support (e.g., psychological assessment, signposting to relevant services, and/or psychological therapy) at any point following discharge.

The available data from 2021 suggest that approximately 127 adult patients were admitted to the ward. The psychology team received approximately 13 referrals for follow-up support via the MDT, 19 via outpatient scar clinics, and 4 self-referrals. The low level of adult self-referrals is interesting as mental health difficulties are prevalent and enduring following a burn injury [9]. It is unclear how many, and why, patients are not self-referring. Given that untreated psychological distress in this population can influence physical recovery [2], it important to ensure patients have access to appropriate psychological care. Therefore, there is a need to understand patient perspectives of psychological support at the burn’s unit, especially in the context of healthcare received from admission to outpatient rehabilitation support. This will help the psychology team make feasible changes to improve the patient experience and facilitate access to appropriate care in line with psychological needs.

### 1.2. Study Aims

To the author’s knowledge, there is no existing study qualitatively exploring the barriers and facilitators to accessing psychological support following a burn injury. This project had the following aims:

1. To understand patient’s perceptions of psychological support and the facilitators and barriers to accessing this.
2. To identify any patient-reported gaps in psychological care during outpatient rehabilitation.
3. To develop practical and feasible service recommendations to improve patient experience, reduce barriers, and enable patients to access the psychology team.

## 2. Methods

### 2.1. Design and Participants

This study employed a qualitative descriptive design to gain an understanding of the lived experiences of burn patients [23]. Thematic analysis was used to identify key topics in the data [24]. Purposive sampling was used to recruit participants who had previously experienced a burn injury to limit sample heterogeneity. Participants were required to be 18+ years old, not receiving psychological support, and under the care of the burns team. Patients attending outpatient clinics in the service were screened for eligibility by AC and LM and were contacted via phone to participate. An information sheet and consent form were sent to a participant’s email. Recruitment commenced in October 2022 and demonstrated 28% uptake (*n* = 11/39). To reach saturation, the authors aimed to conduct 9–17 interviews [25]. All participants were required to give verbal or written consent.

### 2.2. Procedure

A semi-structured interview schedule was developed with reference to relevant burns and psychological literature and discussions with Clinical Psychologists. The interview schedule was reviewed by two service users to check for question comprehension and to reduce significant emotional distress. The interviewer explained the role of psychology to all participants prior to the interview taking place. Interviews were conducted remotely via video-call software or via the telephone between October 2022 and March 2023 and lasted between 35 and 70 min. All interviews were audio recorded, transcribed verbatim, and anonymised. Participants were debriefed following the interview and signposted to services if appropriate.

### 2.3. Ethics

This project was approved by Buckinghamshire NHS Trust, falling under the remit of service evaluation.

### 2.4. Data Analysis

Analysis comprised six phases [24] (See Table 1). Initially, 366 codes were identified, which were then collapsed with overlapping codes to form 12 potential themes, before a final thematic structure to represent study findings was decided.

### 2.5. Trustworthiness or Methodological Rigor

Several checks were carried out throughout the research process to ensure credibility, transferability, confirmability, and dependability. MH and AC individually reviewed a sample of the transcripts to support reflection on codes and themes. Tables were used to store data and were reviewed by MH and AC. A reflective journal was kept by LM throughout the research process.

**Figure 1:**
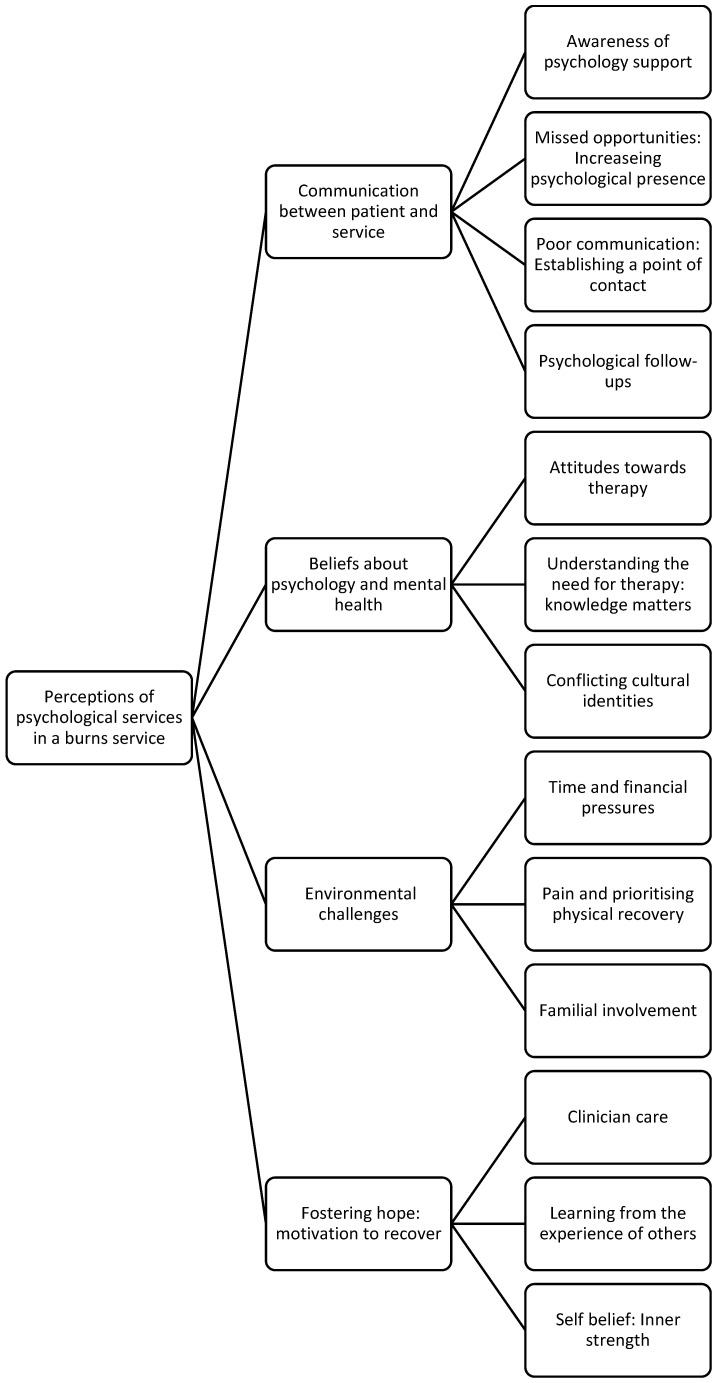
Thematic map of data.

## 3. Results

### 3.1. Sample Characteristics

The sample consisted of 11 participants, 6 females and 5 males, aged 18–70 (see Table 2). All participants were under the care of the burns team but were not currently accessing psychological support. All but one of the participants’ burn injuries occurred in the past two years, with one participant’s burn injury occurring ten years+ ago. All patients burn injuries were below the level of 40% total body surface area.

### 3.2. Overview of the Themes

The analysis identified four overarching themes: (1) ‘Communication between patient and service’; (2) ‘Environmental challenges’; (3) ‘Beliefs about mental health and treatment’; and (4) ‘Fostering hope: motivation to recover’. Within these overarching themes, fifteen sub-themes were identified (see Figure 1). The themes and sub-themes will be highlighted using direct quotations in text. Shortened quotations are indicated by […] and additions for clarity are indicated by (…). Guidelines around quantifying language have been adhered to [26].

#### 3.2.1. Communication between Patient and Service

This theme concerned participants’ experiences of communication with clinicians and psychologists and how such experiences influenced their perception and awareness of psychological services. The identified sub-themes were recognised as potential barriers or facilitators, and each is described below.

##### Awareness of Psychological Support

Some participants reported a lack of awareness of the psychological support available to them following their burn injury, and this was highlighted as a contributing factor as to why patients did not seek psychological support. Participants commonly either themselves stated that awareness was a barrier, or said this is what they believed would have influenced other patients:

*‘Maybe people didn’t know about it, I didn’t know about it. Maybe people don’t know they have right to something’*

(P5).

Some participants recalled that despite being visited by a psychologist, the initial shock of the incident meant it was difficult to engage with information provided by the psychologist at this time:

*‘As far as I can remember when I was in hospital, they did talk to me about it but I was really like out of it on drugs a lot of the time, but I do remember them mentioning it…’*

(P1).

Most participants highlighted that ‘increasing awareness’ of psychological support would improve access. Suggestions included ‘advertising more on clinic boards’ (P4) and distributing information resources. Some participants considered the need for multiple languages and to make information ‘available in different formats for different people’ (P2) to ensure all patients have access. One participant reported that although he was provided with information when discharged, this was ‘information overload’ (P6), and separate information sheets with instructions would have been more accessible.

##### Missed Opportunities: Increasing Psychological Presence

Most of the participants described that patients should be made familiar with the psychology team, *‘So that the patient doesn’t even think about it. They just think this is normal for the hospital’* (P10). Some participants reported that being more familiar with psychological staff during their time on the ward may have encouraged them to seek support following discharge:

*‘I also think that had I been visited by somebody (from the Psychology team) whilst I was in hospital at the time, or maybe a few days before I left, to speak about it, and I had experienced that there and then, then I think I would have been a bit more open to it’*

(P1).

One participant suggested that patients are more likely to be encouraged to seek psychological support by doctors and nurses ‘*because people tend to open up to certain people (clinicians) more, especially when they are interacting with them more, they get more comfortable*’ (P7).

Some participants reported that opportunities to ask appropriate questions about mental health were missed by clinicians:

*‘I was going every Monday to have dressings changed […] No one said to me ‘how are you feeling within yourself?’ ‘Do you want to speak with someone?’*

(P1).

One participant felt psychologists did not ask about ‘*the psychological changes’* (P11), which would have provided an opportunity to establish appropriate support.

##### Poor Communication: Establishing a Point of Contact

Some participants reported difficult experiences trying to access support as an outpatient, including long waiting times and a lack of response following contact made:

*‘I know everyone is overworked. Everyone needs someone right now, so I don’t mind waiting. However, I do need to talk to someone, because otherwise I will let it go as well and I don’t really want to do that?’*

(P8).

Some participants perceived they would ‘*have to go through stress*’ (P11) to contact the psychology team due to past experience with ward communication. Suggestions to improve communication experiences included having a *‘designated person, or place to contact’* (P3) or direct contact numbers to access relevant information.

##### Psychological Follow-Ups

Some participants highlighted that the initial psychology meeting was not enough, with participants attributing this to the shock of the incident, their mind being preoccupied, being on strong medication, or it not feeling like the right time for them:

*‘When it first happens, it sometimes feels like a dream, it feels like another tick box exercise. But your mind is not there, it is thinking about 1000 other things’*

(P7).

Most participants suggested that increasing psychological presence in ‘*follow-up*’ outpatient appointments, ‘*before and after procedures*’ (P10), ‘*dressing changes*’ (P1), and ‘*by doing the active outreach to the patient in intermittent periods of the year*’ (P6), would improve patient experience and facilitate those in need of support to seek help.

#### 3.2.2. Beliefs about Psychology and Mental Health

This theme encapsulated the ways that participants perceived beliefs about mental health and psychology treatment to influence engagement with the psychology team and coping styles. This ranged from psychological stigma towards getting help to a lack of knowledge about psychological therapy and conflicting cultural identities.

##### Attitudes towards Therapy

Participants’ perceptions of therapy, including attitudes towards psychologists and psychological health, were regarded as a barrier to accessing support. Participants spoke of their own attitudes and attitudes they felt other patients might hold. Some participants reported negative beliefs about the usefulness of therapy, ‘*of bringing up trauma time and time again*’ (P1) and *‘revisiting bad memories’* (P6).

One participant expressed concerns about stigma towards seeing a psychologist, stating that some people will ‘*see the term psychology and not go any further’* (P10). Other participants reported that hearing about others ‘*negative experiences’* (P4) of therapy has influenced their own views of psychological support:

*‘My sister […] she tried therapy many different times with different people and all it ever did was bring up bad memories… and she stopped. So, I am drawing on that, I understand why people wouldn’t want to’*

(P1).

Attitudes towards seeking support were identified as another barrier, with some participants assuming that the NHS is too busy:

*‘I know there are a lot of people out there that needs support, but with the amount of people there just aren’t enough doctors or therapists out there to keep up with it’*

(P8).

Others perceived the act of seeking psychological support as conflicting with their own characteristics:

*‘I don’t think I’m made that way. I way quite a private person’*

(P1).

*‘I personally wouldn’t go because I like to handle things myself sometimes’*

(P8).

##### Understanding the Need for Therapy: Knowledge Matters

Some participants identified barriers to accessing support including a lack of education about what a psychologist does and what improvements can be made from therapy:

*‘I think it’s a lack of education from a regular patient not understanding that a clinical psychologist is someone who just walks at your own pace’*

(P6).

Other participants highlighted that providing information/education about psychology and what it achieves would improve access to psychological support:

*‘People don’t really know what you do at times. […] Can you expand on it and describe it to people?’*

(P10).

*‘I think, having all the information on one page, so you could make a judgement for yourself, whether it’s suitable for you or not’*

(P3).

##### Conflicting Cultural Identities

Some participants highlighted the importance of understanding how cultural frameworks shape patient interactions with healthcare systems and professionals. Participants spoke about their own cultural dilemmas in this context:

*‘I grew up in an Asian Indian household. But where I have grown up in the British culture, things are changing. Mental health is being talked about more, but it’s still two sides in my head … of the little argument. Most of the time, the house I grew up with wins’*

(P8).

Some participants reflected on how cultural values may influence access to support in the context of gender and class:

*‘Women are not always given equal rights and not treated equally. If the scar is somewhere, in a sensitive area ’ … ’ maybe the husband might not like the person going to get emotional support…’*

(P4).

*‘I came from a working-class background and its actually my accident that made me go to university. So, it’s a traditional thing of ‘you never talk about it’*

(P10).

Some participants described how their expression of pain is influenced by their culture:

*‘When it comes down to pain… do not show it to your family members. I am Indian, so I don’t want to show it to my nieces or whatever’*

(P9).

#### 3.2.3. Environmental Challenges

This theme encapsulates the environmental factors that participants perceived to impede access to psychological support. These challenges ranged from time and financial pressures to familial involvement.

##### Time and Financial Pressures

Most participants highlighted practical pressures associated with time availability, finances, and the location of the hospital that could influence an individual’s opportunity to attend psychological support:

*‘People are living their own lives; they don’t have time for these things’*

(P5).

*‘I have already taken time for a scan for about two hours. so I feel that my job is being affected’*

(P4).

Most participants suggested that remote therapy ‘via *(MS) Teams’* or *‘phone’* would improve access to psychological support.

##### Pain and Prioritizing Physical Recovery

Some participants highlighted physical pain as a barrier to engaging with psychological support whilst on the ward and following discharge:

*‘It was just like, all the people meeting me and saying they are a team… but because of the pain, my inclination was just like ‘can they change this and let me go home’*

(P11).

Some participants highlighted the desire to get back to life as normal, prioritizing physical recovery over psychological appointments:

*‘You kind of put any kind of psychological part of your journey on the back feet, because I just wanted to get back to a physical condition where I could carry on with life as normal…’*

(P6).

*‘It was literally week to week, getting my next repair, planning the next, seeing the result and planning what’s the next one’*

(P10).

##### Family Involvement

Some participants reported that having close family support was a strong protective factor and negated the need for psychological help following their injury:

*‘I have great family; I have people I can talk to if I need to’*

(P7).

One participant highlighted that without family support, she would have been encouraged to seek psychological help:

*‘If I didn’t have that I would have probably needed someone to check in with me, like once a week, or something like that?’*

(P8).

Others perceived familial views to be a barrier to access, especially if family members disapprove and are responsible for patient transport:

*‘People have wounds, so they have to get help from the members of the family because if they are not mobile and all that, it is difficult’*

(P4).

#### 3.2.4. Fostering Hope: Motivation to Recover

This theme encapsulates participant experiences of fostering hope following a burn injury. Participants reported that reassurance from empathic clinicians, access to recovery stories, and inner strength facilitated hope and influenced attitudes towards psychological support.

##### Clinician Care

Some participants revealed that their experience on the ward was impacted by the empathy received from clinicians:

*‘I was screaming I was in so much pain, and I was getting back into bed, he said ‘you need to try and be braver’ […] so to have a comment like that, was quite devastating’*

(P1).

*‘Majority if not all the people I met were just how you would expect them to be. They were lovely, they helped me though treatment’*

(P8).

Some participants felt that clinicians could encourage positivity and that there was a *‘light at the end of the tunnel’* (P7) through providing reassurance to patients and families:

*‘(If clinicians said) ‘Yes, we understand how bad it is, but it can get better, and that’s where you should strive for… yes, it is bad and you probably think it couldn’t be worse, but it can get better’’*

(P7).

*‘If a psychologist said ‘listen we have seen a lot of people go through this, and they cope later, things become normal. Don’t worry you will cope’ […]. That makes the person come back and relax, they think ‘OK I can cope myself’*

(P11).

##### Learning from the Experience of Others

Some participants described how having access to other patient’s recovery would have reduced their sense of isolation and increased their motivation to seek psychological support:

*‘If on that patient information sheet that’s provided, there was […] some feedback from an actual patient on there […] Just something where it’s got another who’s giving their experience, and that they actively sought (support) with Psychology team afterwards and what it did for them’*

(P6).

This was further illustrated by some participants who reported that witnessing others’ healthcare experiences influenced their own coping style:

*‘I’ve seen how patients cope and whatever. So, I just took it myself, I said ‘wow some people can cope with the injuries they have… why can’t I?’*

(P11).

##### Self-Belief: Inner Strength

Most participants described a sense of self-belief following their injury. Some participants reflected on their personal characteristics that they used to overcome burn-related difficulties:

*‘I am quite strong willed and have a strong character, and I did prove that when this happened to me’*

(P7).

Others described personal determination and the flexible *‘mindset* (P10)’ required to overcome their burn injury:

*‘For me I never wanted it to be the end point, I didn’t want this to be the only thing I was remembered for—for having this terrible accident’*

(P10).

Some participants reflected on how they chose to positively reframe their experience, which enabled them to adjust without psychological support:

*‘Just being positive. Shining a light on what good could come out of it, […] as long as you lean towards the positive side of things. I did that for myself, and I know it has helped me’*

(P7).

## 4. Discussion

This qualitative study explored the facilitators and barriers to psychological support following a burn injury. The aim was to understand patient perceptions of available psychological support, identify any gaps in psychological care, and develop feasible service recommendations. Four main themes highlighted how patients’ perceptions of access to psychology were influenced by patient and service communication, beliefs about mental health, environmental challenges, and fostering patient hope.

In line with the COM-B model [12], previous research has demonstrated that a lack of ‘awareness’ about psychological services reduces an individual’s capability to access appropriate care [27,28]. The participants in this study felt that due to the trauma of sustaining a burn injury, the initial psychological capability to engage with, and retain, information about psychological services whilst on the ward is low. The NBCS recommend patients receive a psychological screen as soon as clinically appropriate and prior to discharge [21]. The initial psychosocial screening has been evidenced as a useful tool for the identification of early psychological difficulties [22,29]. However, there is acknowledgement that psychological difficulties fluctuate post-injury [30]. Therefore, there may be value in implementing follow-up psychosocial screenings at multiple stages of a burn patients’ treatment [11,31]. One study found that the presence of psychologists at outpatient appointments enabled the identification and treatment of burn-related psychological concerns that were not met earlier in the treatment pathway [32]. Consistently, most participants in this study suggested that increasing psychological presence following discharge and during outpatient appointments would facilitate access.

Patient beliefs about mental health and treatment were perceived by participants to influence their access to psychological support. It is widely acknowledged that there is a stigma related to mental illness and treatment [33,34] and that this stigma acts as a motivational barrier to professional support [35]. Consistent with cognitive theories of PTSD, it is understandable that those who fear ‘re-traumatisation’ through therapy choose to avoid psychological services [16]. Participants perceived that increased education and information resources about mental health and treatment would reduce this barrier and increase motivation to engage with support. This is supported by effective anti-stigma interventions aimed at improving mental health utilization in the community [36].

The Cultural Determinants of Help Seeking Model (CDHSM) describes how individual health behaviours (e.g., the expression of pain, choice to seek professional help, or self-reliance) are determined by (1) beliefs about disease cause and curability; (2) anticipated response from others; and (3) social support, burdens, and constraints [19]. In line with this, participants described a series of cultural dilemmas faced by burn patients receiving care (e.g., the disclosure of pain, seeking psychological support as female with a shame-inducing injury, and seeking psychological support whilst endorsing traditional masculine norms). A body of literature has evidenced the failure of some psychological services to meet the needs of individuals from global majority backgrounds [28,37]. Alongside the provision of culturally appropriate resources, developing clinician awareness of some of the cultural determinants of healthcare behaviours is vital for ensuring that patients receive the best care. ‘Cultural humility’ is the process of developing awareness of one’s own implicit biases, as well as of the cultural diversity of others [38]. A recent case study outlined that the dissemination of psychological skills falls within the remit of a clinical psychologist working in a Burns MDT [29]. Therefore, psychologists are in the unique position to promote cultural humility through the facilitation of supervision and staff training.

As outlined in past studies, opportunity barriers to psychological support include prioritising physical recovery, time, geographical, and financial pressures. The promotion of remote therapy was regarded by participants to reduce these barriers. Importantly, remote therapy is found to be effective and feasible for PTSD [39]. Familial support was considered by most participants to influence patient recovery. A recent review found social and familial support to facilitate resilience and recovery in burn patients [40]. In line with the CDHS model, the anticipated response from family members, as well as environmental constraints (e.g., family members willing to support patient to travel to appointments), might also prevent a patient from accessing psychology support [19]. Engaging and collaborating with family members was highlighted by some participants as an important way to increase opportunities for patients to access psychological care, an approach also recommended in the literature [41].

Most participants reflected on the personal characteristics required to cope with their injury, including hope, having a positive mindset, and inner strength. Such characteristics are in line with the concept of post-traumatic growth (PTG), which indicates positive psychological change beyond pre-trauma functioning, beliefs, and values [40]. Participants perceived factors such as clinician care and others’ recovery stories to facilitate hope and motivation to engage with psychology. The experience of receiving empathy (and/or a lack of) and reassurance from clinicians was regarded by most participants as significantly influencing their experience. This is in line with recent studies that found that empathic care from clinicians improved outcomes during therapy [42]. NBCS recommend all MDT members are trained to recognise and respond appropriately to psychological distress [21]. There is scope for the psychology team to provide training to clinicians supporting patients in this context. In addition, some participants outlined the importance of fostering hope and positivity through access to patient recovery stories. Past research has highlighted the value of peer support and shared experiences following a burn injury [43]. The provision of booklets with patient stories has been found to be a useful tool for positively reframing the perspectives of burn patients [44]. Access to patient recovery stories through information resources may therefore provide a feasible opportunity for the service to motivate burn patients towards psychological recovery.

### 4.1. Service Recommendations and Rationale

Recommendations were made based on the study findings (see Table 3). The findings were distributed to a psychology team and feedback was provided.

### 4.2. Limitations and Future Research

Although this study has a relatively small sample size, it is appropriate for an exploratory qualitative study [45]. The aim of qualitative research is to better understand participant experience and not achieve generalisability. Various aspects of this study were subjective, making it vulnerable to several biases. First, the data itself were vulnerable to interpretation bias, despite several validation and credibility checks. As with all voluntary research, self-selection bias was likely, as only 28% of participants contacted chose to contribute [46]. Social desirability bias may have influenced participant responses due to researcher presence. Finally, the generalisability of findings was limited by the heterogeneity of participant characteristics (e.g., the severity of the burn injury).

Despite these limitations, this study provides opportunities to conduct more nuanced investigations in this field. First, it might be useful to investigate whether factors such as ‘time since burn injury’, and ‘scar severity’ influence participants’ experiences of burn outpatient care. Second, culture was identified as a key influence on patient experiences with psychological care, yet cultural considerations in the post-burn adjustment literature remain understudied [1]. Future research could systematically investigate the relationship between clinician cultural humility and patient experience in burns settings. This would further contribute to the shaping of guidance relating to psychological support in burn services.

## 5. Conclusions

This qualitative study aimed to explore the barriers and facilitators to psychological support in a burns unit from a patient perspective. Providing psychoeducation about psychology and increasing psychological presence throughout rehabilitation offers the opportunity to facilitate access. Given the service context, it is not feasible for a psychologist to be present at every patient’s medical appointment. However, if every intervention a patient receives from the point of burn injury ‘influences their scar worn for life’, then focus must be on ensuring that the workforce is psychologically informed and empathic [47,48]. Identifying psychological distress and responding appropriately may well minimise negative psychological impacts throughout patient recovery. Further findings stressed the value of promoting the practice of cultural humility among staff supporting patients in this context. Improving access to culturally appropriate resources and signposting information will offer more opportunity for clinicians to establish a collaborative and patient-centred approach in burns care.

## Figures and Tables

**Table 1:** Stages of data analysis.

**Phase**	Overview
1	Each interview was listened to and transcribed. Initial notes outlining ideas were made and all transcripts were saved using NVivo V.12.
2	Features of the entire data set were systematically coded. Data were matched to each code when appropriate. At this point, 366 codes were identified.
3	Codes were collated into emerging themes (*n* = 26). All data relevant to each theme was gathered.
4	Themes were reviewed in the context of the collected codes and entire data set. Two clinical supervisors were consulted.
5	Overarching themes (*n* = 4) and sub-themes (*n* = 15) were defined and refined in the context of the entire data set. These themes were discussed with clinical supervisors AC and MH.
6	Final theme and codes were written up. A thematic map was constructed (See Figure 1). Relevant excerpts from the data were selected to demonstrate codes/themes.

**Table 2:** Participant sample characteristics.

Participants		
Sex	Male	5 (45%)
	Female	6 (55%)
	Other	0
Age	21–30	2 (18.2%)
	31–40	4 (36.4%)
	41–50	0
	51–60	4 (36.4%)
	61+	1 (9.1%)
Ethnicity	White/White British	3 (27.3%)
	Asian/Asian British	7 (63.7%)
	Black/African/Caribbean/Black British	1 (9.1%)
	Mixed	0
	Other (inc. Arab)	0

**Table 3:** Recommendations and rationale for the burns psychology team.

Recommendations	Rationale
Recommendations to improve communication and increase awareness of psychological support	
- Service to provide resources such as booklets and leaflets with information about the psychology team. - Resources should include psychology teams contact details. - Resources should be distributed as handouts or via email and separate from the burn information pack.	Participants identified that a lack of knowledge and awareness of psychological support reduced their capability to self-refer. Suggestions were made to improve the accessibility of information given the barriers faced at time of burn injury (e.g., time, physical pain, and medication), including providing printouts of leaflets/handouts and online copies. Participants perceived accessing psychology to be stressful and time-consuming, with less understanding about how to refer to the team directly. Providing direct contact information was suggested by several participants. One participant highlighted that the burn information pack was too dense and that it was difficult to engage with the psychological information.
- Psychologist to continue attending outpatient clinics where feasible and appropriate. Where not feasible, psychologist to support clinicians to identify psychological distress, review mood, and discuss psychological support.	Participants highlighted that often patients might ‘not think’ to seek psychological support due to prioritising physical recovery. As such, it is important for clinicians to increase psychological presence on the ward and via outpatient appointments. Suggestions by participants included a psychologist being present whilst on the ward, for psychologists to be present in dressing change appointments, and for nurses and doctors to review the mood of patients and recommend them psychological support.
- Psychology team to provide clinicians with psychology resources (booklets/information handouts) for patient and their families.	
- Resources made available on the ward and in clinical settings for families and patients to view.	
- Psychologist to offer routine follow-up calls following discharge to inform patients of support available and identify any psychological distress.	Participants highlighted that psychology reviews on the ward may be difficult to engage with due to physical barriers (e.g., shock, pain, and medication). Furthermore, participants stated that the longer a patient is away from the ward, the less likely they are to want to revisit and seek support (e.g., due to fear of revisiting trauma or the perception that the service is too in demand). Follow-ups were recommended to ensure patients are aware of the service and therapies available. It was suggested that follow-up calls may prompt patients to think about their psychological needs.
Recommendations to engage with different beliefs about psychology and mental health	
- Resources should include psychoeducation about support and therapy offered by psychology team (what it does and how it works) and psychoeducation about mental health following a burn injury. - Resources used should be cocreated with service users to ensure acceptability across a range of patient needs, e.g., language. - Information should be made available for family and friends of the patients. Culturally adapted resources and signposting information should be provided.	Participants identified that stigma resulting from negative stories, fear of re-traumatisation, cultural conflicts, and lack of knowledge about mental health, mental health therapies, and psychologists may reduce referrals to the psychology team.
Recommendations to reduce practical barriers to accessing psychological support	
- Psychologists to ensure that patients have information about remote therapy opportunities (via resources).	Participants identified that practical barriers, such as travel time, work, finances, and physical pain, may also prevent a patient contacting the psychology team.
- Resources to include information for family members about psychological therapy.	
Recommendations to encourage and foster a sense of hope and motivation whilst under the care of the Burns MDT	
- Service to ensure that resources (booklets/information handouts) include some examples of positive experiences and stories from patients recovering well who accessed psychological support.	Participants highlighted that an integral part to recovery is fostering a sense of hope. Allowing access to past patient stories and experiences of using psychology would facilitate hope and motivation to engage in therapeutic support if appropriate. This was highlighted as important for patients and their families.
- Service to ensure that resources include signposting information for peer support and alternative services.	

## Data Availability

Not applicable.
